# Disparities in knowledge, attitude, and practices of infection prevention and control of Lassa fever among health care workers at The Federal Medical Centre, Owo, Ondo State, Nigeria

**DOI:** 10.11604/pamj.2021.38.357.26208

**Published:** 2021-04-14

**Authors:** Victor Okoliko Ukwenya, Temiloluwa Adeola Fuwape, Tokunbo Ibukun Fadahunsi, Olayinka Stephen Ilesanmi

**Affiliations:** 1 Department of Human Anatomy, School of Health and Health Technology, Federal University of Technology, Akure, Nigeria,; 2 Department of Global and Community Health, College of Health and Human Services, George Mason University, Virginia, United States of America,; 3 Volgenau School of Engineering, George Mason University, Virginia, United States of America,; 4 Department of Community Medicine, University College Hospital Ibadan, Oyo State, Nigeria

**Keywords:** Lassa fever, prevention, knowledge, health workers, Nigeria

## Abstract

**Introduction:**

the knowledge and practices on Lassa fever (LF) infection prevention and control (IPC) remains poor among health workers in Nigeria despite LF endemicity. This study aimed to evaluate the knowledge, attitude, and practices of healthcare workers at the Federal Medical Centre, Owo towards LF.

**Methods:**

this was a cross-sectional study among 451 healthcare workers who were enrolled using a simple random sampling technique. Data were collected using a semi-structured interviewer-administered questionnaire and analyzed with SPSS version 23. Adequate knowledge, positive attitude, and good practice of LF infection, prevention, and control were determined by the proportion of respondents who scored >80% in each category. Descriptive statistics were done. Associations were explored using Chi-square tests.

**Results:**

the mean age of respondents was 37.95±8.43 years, and 169 (37.5%) were doctors. The mean overall knowledge score was 18.33±2.14, and 236 (52.3%) had appropriate knowledge, 109 (24.2%) had a positive attitude, while 351 (77.8%) demonstrated adequate preventive practices towards LFIPC. Laboratory scientists had five times the odds of appropriate knowledge of LF IPC (OR=4.886; 95%CI: 1.580-15.107). Pharmacists had ten times odds of positive attitude towards LF IPC (OR=10.093; 95%CI= 1.055-95.516). Pharmacists had nine times odds of good LF IPC practices (OR=8.755; 95%CI=1.028-74.531).

**Conclusion:**

disparities in knowledge, attitude, and practices of LF IPC exist among healthcare workers. To strengthen IPC, intervention strategies like training to address such gaps are needed.

## Introduction

Lassa Fever (LF) is a zoonotic disease caused by the Lassa virus. Lassa fever is named after Lassa town in Nigeria where it was first isolated. Humans contract the virus mainly through contact with infected excreta of *Mastomys natalensis* rodents (commonly known as Multimammate rats), which is a natural reservoir for the virus and are ubiquitous in the country [1]. Infected rodents are reservoirs capable of excreting the virus through urine, saliva, excreta, and other body fluids to humans [2]. Secondary transmission of the virus between humans occurs through direct contact with infected blood or body secretions [3]. It happens mainly in doctors caring for patients, although anyone who comes into close contact with a person who carries the virus is at risk of infection. The incubation period for LF is 1-3 weeks [4]. Lassa fever is known to be endemic in Benin, Ghana, Guinea, Liberia, Mali, Sierra Leone, Togo, and Nigeria [5]. Lassa fever is endemic in Nigeria and cases are recorded all year round [6]. This contributes largely to the risk of spread that occurs in Nigeria and other countries with similar ecological factors [1]. Three states contribute about 95% of the LF positive cases in Nigeria, namely Edo, Ondo, and Ebonyi [7].

Nosocomial infection is a hospital-acquired infection that occurs among healthcare workers (HCWs), to patients or patient relatives through contact with contaminated beddings or secretions [6,8,9]. The control of hospital infection is very important and could be achieved through positive knowledge, attitude, and practice towards LF infection [10]. Positive medical attitude and practice will help to reduce morbidity and mortality resulting from LF [5,9]. Some studies identified that index cases of LF are usually from the community but LF outbreaks have been significantly related to hospital transmission [8,11]. Evidence abounds that LF symptoms and signs are indistinguishable from febrile diseases such as malaria and other viral hemorrhagic fevers such as Ebola [11,12]. Approximately 80% of the symptoms are mild and such go often undiagnosed [1]. Death can occur within two weeks of the onset of LF symptoms due to multi-organ failure [5]. Since LF presents with no specific symptoms, clinical diagnosis is often difficult especially at the onset of the illness [1]. Accurate diagnosis of LF is enabled by differential laboratory testing, clinical manifestations, epidemiological findings since definitive diagnosis requires investigations available only in highly specialized laboratories [1]. Prevention of transmission is of utmost importance in the control of the spread of LF, hence the universal precautions that have been outlined to protect health workers from contracting the infection [13].

Infection prevention and control (IPC) has been described as a concept which aims at containing disease transmission either in health facilities or in communities [14]. In the LF context, adherence to IPC measures such as the use of goggles, full-body PPE, face masks/face shields, boots, aprons, and gloves have been validated as a vital component of strategies for the control of potential outbreaks of LF [15]. It has been reported that a major contributor to hospital-acquired LF infection is the poor knowledge of LF, and poor knowledge of IPC measures among healthcare workers [14]. This occurrence is however unfortunate because HCWs are active agents of promoting compliance to disease preventive measures. If HCWs are then lacking in IPC for LF, how then could community members be empowered with sufficient health education on IPC measures needed to tackle LF? Hence, healthcare settings are potential sites for sporadic outbreaks of LF infection. In some instances, doctors have been reported to have displayed higher IPC practices than other groups of HCWs [15]. It, therefore, becomes pertinent to assess the disparity (if any) in the knowledge, attitude, and practices of LF IPC among HCWs. Lassa fever is endemic in Ondo State [5] yet the knowledge, attitude as well as preventive practices with respect to it have been reported to be poor [16]. This study therefore aimed to assess the knowledge, attitude, and practices of HCWs at the Federal Medical Centre, Owo towards LF IPC.

## Methods

**Study design:** a descriptive cross-sectional design was employed.

**Study area:** Federal Medical Centre, Owo. It is a tertiary health institution that provides primary, secondary, and tertiary levels of healthcare. It provides healthcare services to the residents of Ondo state, and neighboring states including Kogi, Edo, Ekiti, and Osun States. The Infection Control Ward (ICW) was created in January 2017 solely for the management of LF infection. It was renamed the LF Infection Control and Research Centre (ICRC) after the unprecedented outbreak of LF disease in the first few months of 2018. The ICW which was an 8-bedded ward was upgraded to a 34-bedded facility on 28^th^ February 2018. The ICRC has a collaboration with Nigeria Centre for Disease Control (NCDC), and international organizations for research and management such as the Alliance for International Medical Action (ALIMA) and African Centre of Excellence for Genomics of Infectious Diseases (ACEGID). The activities of the ICRC are controlled through an Emergency Operations Centre (EOC) that was set up to serve as the command center for all activities during an outbreak. Aside from clinical management of cases, a follow-up clinic exists for discharged patients in the facility.

**Study population:** consenting staff members in clinical departments were studied.

**Sampling methods:** this research used simple random sampling. A list of the clinical staff of the hospital was collected from the Administrative Department. From the available staff list, the units/departments were arranged serially. A table of random numbers was used to select at least one out of every three staff in each unit/department.

**Data collection:** a semi-structured, interviewer-administered questionnaire was used which consisted of four sections; section A: sociodemographic characteristics; section B: knowledge of HCWs towards infection prevention and control; section C: attitude of HCWs towards infection prevention and control; section C: practices of HCWs towards infection prevention and control.

**Data management:** questionnaires were checked for omissions and errors after collection and correction were made where necessary. Data were analyzed with the SPSS version 23. The data were summarized using mean and standard deviation for continuous variables, frequencies, and percentages for categorical variables. Knowledge, attitude, and practice scores were computed with “+1” assigned for correct response and “0” assigned for incorrect response. The maximum score possible for the knowledge section was 23, while attitude was 6 and practice was 20. Appropriate knowledge, positive attitude, and good practice were assigned scores >80% while inappropriate knowledge, negative attitude, and poor practice were assigned to scores ≤80%. These scores were graded using Bloom´s cut-off point. We conducted bivariate analysis on the sociodemographic characteristics of respondents and their levels of knowledge, attitude, and practices regarding LF IPC using Chi-square tests. The predictors of appropriate knowledge, positive attitude, and good IPC practices regarding LF were performed using Logistic regression analyses. Logistic regression analyses were performed on variables that were significant at <10% in the bivariate results. P-values < 0.05 were accepted as statistically significant.

**Ethical consideration:** informed consent (written/verbal) was obtained from the respondents, who were made to understand that participation was voluntary. Information obtained was kept confidential and there was no consequence for non-participation. Approval for the study was obtained from Health Research Ethics Committee, Federal University of Technology, Akure, Ondo State and Federal medical Centre, Owo (FMC/OW/380/VOL.LXXXIX/165).

## Results

**Figure 1:**
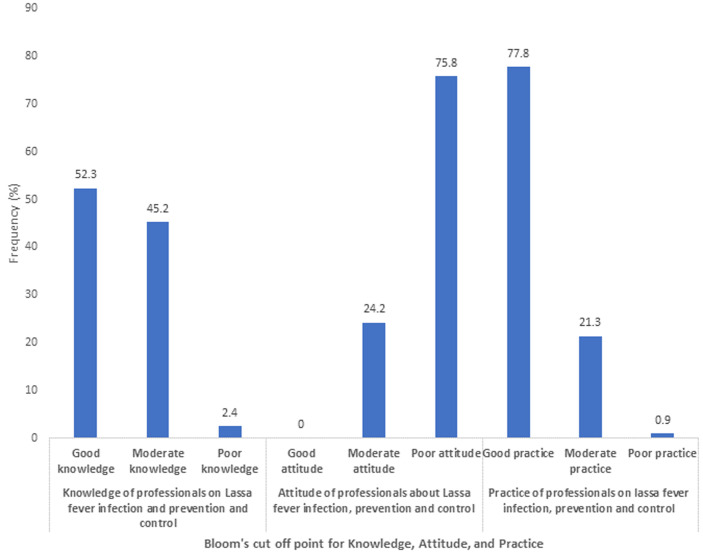
the bloom´s cuff point for knowledge, attitude and practice of health workers towards LF infection, prevention and control

**Figure 2:**
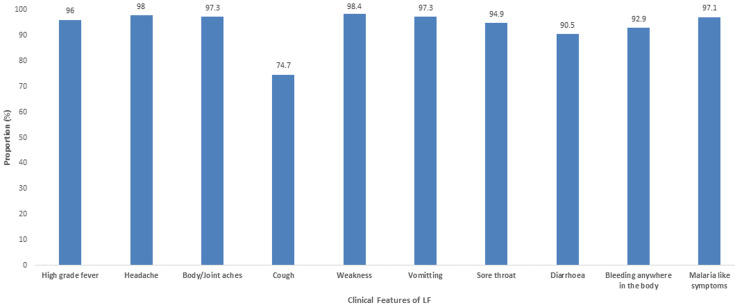
the proportion of health workers that correctly identified the clinical features of LF among health care workers at Federal Medical Centre, Owo 2020

**Figure 3:**
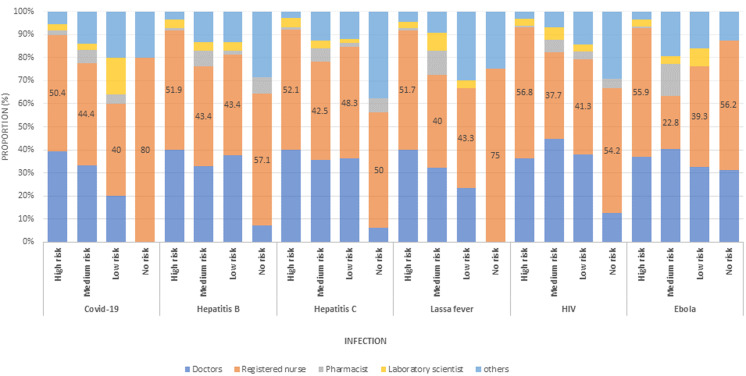
perceived level of risk of contracting infectious disease among health care workers at Federal Medical Centre, Owo 2020

**Figure 4:**
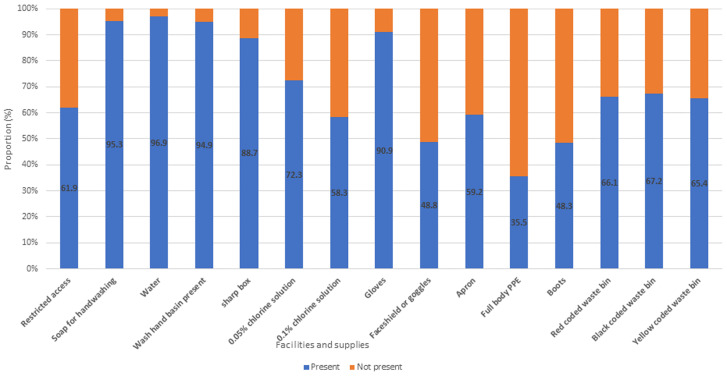
the presence of facilities and supplies when needed by the health workers as precautionary measures

Table 1 shows the sociodemographic characteristics of the respondents. The mean overall knowledge was 18.33±2.14. Of the 451 respondents, 270 (59.9%) were females. The mean age of respondents was 37.95±8.43 years. Also, 169 (37.3%) were doctors, and 117 (25.9%). More than half of the respondents had appropriate knowledge on LF infection, prevention and control (52.3%); 24.2% had a positive attitude while 77.8% demonstrated good preventive practice towards LF. Most of the respondents (96.5) had training on standard precautions as depicted in Table 2. According to the Bloom´s cut-off point as shown in Figure 1, 52.3% of respondents had appropriate knowledge (Score of >80%), while 75.8% had a negative attitude (Score of <60%) and 77.8% had adequate preventive practices towards LF IPC. The majority of the respondents correctly identified the clinical features of LF as depicted in Figure 2. About 96% of respondents identified high-grade fever as one of the clinical features of LF while 94.9% correctly identified sore throat as a feature. Among those who reported no risk of contracting LF, 75% were nurses, while among those who qualified their risk of contracting the Lassa virus as high, 40.1% were doctors (Figure 3). The presence of facilities and supplies when needed as precautionary measures are shown in Figure 4. In all, 430 (95.3%) had access to and used soap for handwashing when needed, while 160 (35.5%) had full PPE when needed. About two-thirds had access to red 298 (66.1%), black 303 (67.2%), and yellow 295 (65.4%) colour-coded waste bins when they were needed.

**Table 1:** socio demographic characteristics of respondents among healthcare workers of Federal Medical Centre, Owo, Ondo State, Nigeria

Variables (n=451)	Frequency	%
Age of respondents, M±SD	37.95±8.43	
Year of practices professionals, M±SD	11.19±7.63	
Age		
≤30 years	117	25.9
31-40 years	168	37.3
41-50 years	128	28.4
51-60 years	38	8.4
Sex		
Male	181	40.1
Female	270	59.9
Religion		
Christianity	420	93.1
Islam	31	6.9
Ethnicity		
Yoruba	379	84.0
Ibo	34	7.5
Hausa	2	0.4
Others^#^	36	8.0
Profession		
Doctor	169	37.5
Registered nurse	224	49.7
Pharmacist	10	2.2
Laboratory scientist	16	3.5
Others^*^	32	7.1
Highest educational qualification		
PHD	2	0.4
Postgraduate	31	6.9
Tertiary	418	92.7
Department		
Adult accident and emergency	18	4.0
Infectious ward/Community Health	42	9.3
Diagnostic (laboratory and radiology)	47	10.4
Obstetrics and gynaecology	36	8.0
Paediatrics	42	9.3
Medicine (family medicine, medicine, staff clinic)	74	16.4
Surgery (orthopaedics, ENT, ophthalmology)	74	16.4
Other clinicals (anaesthesia, dental, psychiatry, pharmacy, physiotherapy, house officer)	118	26.2

# Urhobo, Tiv, Igbira, Ogoni, Itsekiri ^*^Radiographer, radiologist, pharmacy technician, community health extension worker, physiotherapist, laboratory technician, dental technician, nutritionist

**Table 2:** the level of knowledge, attitude and practice of health workers towards Lassa infection, prevention and control with training on standard precaution

Variables (n=451)	Frequency	%
Overall knowledge, M±SD	18.33±2.14	
Knowledge of health workers		
Appropriate knowledge	236	52.3
Inappropriate knowledge	215	47.7
Attitude of health workers		
Positive attitude	109	24.2
Negative attitude	342	75.8
Practice of health workers		
Adequate practice	351	77.8
Inadequate practice	100	22.2
Had trainings on standard precautions		
Yes	435	96.5
No	16	3.5

We found significant relationships between age, sex and profession of respondents and level of knowledge of LF infection, prevention and control (χ^2^= 13.802, p= 0.003), (χ^2^= 23.654, p= <0.001), (χ^2^= 49.434, p= <0.001) respectively. Also, a significant association existed between sex, and department and attitude of respondents towards LFIPC ((χ^2^= 4.782, p= 0.029), (χ^2^= 14.385, p= 0.045) respectively), but profession was not significantly associated with the attitude (χ^2^= 8.481, p= 0.075). We found significant associations between age (χ^2^= 23.469, p= <0.001), sex (χ^2^= 11.820, p= 0.001), profession (χ^2^= 31.159, p= <0.001) and department (χ^2^= 24.161, p= 0.001) of health workers towards LF IPC (Table 3). Individuals aged 41-50 years had two times odds of having appropriate knowledge of LF IPC compared to others (odds ratio (OR)= 1.766; 95% confidence interval (CI)= 1.011-3.085). Likewise, nurses had two times the odds of appropriate knowledge of LF IPC (OR= 2.392; 95% CI: 1.405-4.072), while laboratory scientists had five times the odds of appropriate knowledge of LF IPC (OR= 4.886; 95% CI: 1.580-15.107) compared to doctors (Table 4). Doctors had three times odds (OR= 2.654; 95% CI= 1.029-6.845) and pharmacists had ten times odds (OR= 10.093; 95%CI= 1.055-95.516) of positive attitude towards LF IPC compared to other healthcare workers (Table 5). Doctors had eight times the odds of good LF IPC practices compared to others (OR= 8.282; 95%CI= 1.670-41.065). Pharmacists had nine times odds of good LF IPC practices (OR= 8.755; 95% CI= 1.028-74.531) compared to others (Table 6).

**Table 3:** association between sociodemographic and level of knowledge, attitude and practice of health workers towards LF infection, prevention and control

Sociodemographic characteristics (n=451)	Knowledge		Chi-Square (χ^2^) and p-value	Attitude		Chi-Square (χ^2^) and p-value	Practice		Chi-Square (χ^2^) and p-value
	Appropriate Knowledge n (%)	Inappropriate knowledge n (%)		Positive Attitude n (%)	Negative attitude n (%)		Good Practice n (%)	Poor Practice n (%)	
**Age (years)**									
≤30	76 (65.0)	41 (35.0)	χ^2^=13.802	30 (25.6)	87 (74.4)	χ^2^=6.254	74 (63.2)	43 (36.8)	χ^2^=23.469
31-40	89 (53.0)	79 (47.0)	p= 0.003	38 (22.6)	130 (77.4)	p= 0.100	132 (78.6)	36 (21.4)	p= <0.001
41-50	56 (43.8)	72 (56.2)		26 (20.3)	102 (79.7)		113 (88.3)	15 (11.7)	
51-60	15 (39.5)	23 (60.5)		15 (39.5)	23 (60.5)		32 (84.2)	6 (15.8)	
**Sex**									
Male	120 (66.3)	61 (33.7)	χ^2^=23.654	34 (18.8)	147 (81.2)	χ^2^=4.782	126 (69.6)	55 (30.4)	χ^2^=11.820
Female	116 (43.0)	154 (57.0)	p= <0.001	75 (27.8)	195 (72.2)	p=0.029	225 (83.3)	45 (16.7)	p=0.001
Religion									
Christianity	222 (52.9)	198 (47.1)	χ^2^=0.685	102 (24.3)	318 (75.7)	χ^2^=0.046	329 (78.3)	91 (21.7)	χ^2^=0.908
Islam	14 (45.2)	17 (54.8)	p=0.408	7 (22.6)	24 (77.4)	p=0.831	22 (71.0)	9 (29.0)	p=0.341
**Profession**									
Doctor	123 (72.8)	46 (27.2)	χ^2^=49.434	31 (18.3)	138 (81.7)	χ^2^=8.481	109 (64.5)	60 (35.5)	χ^2^=31.159
Registered nurse	96 (42.9)	128 (57.1)	p= <0.001	61 (27.2)	163 (72.8)	p= 0.075	190 (84.8)	34 (15.2)	p= <0.001
Pharmacist	4 (40.0)	6 (60.0)		1 (10.0)	9 (90.0)		7 (70.0)	3 (30.0)	
Laboratory Scientist	5 (31.2)	11 (68.8)		4 (25.0)	12 (75.0)		15 (93.8)	1 (6.2)	
Others#	8 (25.0)	24 (75.0)		12 (37.5)	20 (62.5)		30 (93.8)	2 (6.2)	
**Department**									
Adult accident and emergency	10 (55.6)	8 (44.4)	χ^2^=7.240	8 (44.4)	10 (55.6)	χ^2^=14.385	15 (83.3)	3 (16.7)	χ^2^=24.161
Infectious ward and community health	21 (50.0)	21 (50.0)	p= 0.404	7 (16.7)	35 (83.3)	p=<0.045	40 (95.2)	2 (4.8)	p= <0.001
Diagnostic^*^	19 (40.4)	28 (59.6)		10 (21.3)	37 (78.7)		38 (80.9)	9 (19.1)	
Obstetrics and gynaecology	18 (50.0)	18 (50.0)		2 (5.6)	34 (94.4)		32 (88.9)	4 (11.1)	
Paediatrics	22 (52.4)	20 (47.6)		10 (23.8)	32 (76.2)		28 (66.7)	14 (33.3)	
Medicine^**^	39 (52.7)	35 (47.3)		19 (25.7)	55 (74.3)		64 (86.5)	10 (13.5)	
Surgery***	35 (47.3)	39 (52.7)		18 (24.3)	56 (75.7)		53 (71.6)	21 (28.4)	
Other Clinicals^****^	72 (61.0)	46 (39.0)		35 (29.7)	83 (70.3)		81 (68.6)	37 (31.4)	

# Radiographer, radiologist, pharmacy technician, community health extension worker, physiotherapist, laboratory technician, dental technician, nutritionist ^*^Diagnostic department: laboratory and radiology ^**^Medicine: family medicine, staff clinic and medicine ^***^Surgery: orthopaedics, ENT, ophthalmology, surgery ^****^Other clinicals: anaesthesia, dental, psychiatry, pharmacy, physiotherapy, house officers

**Table 4:** multivariate analysis to determine the association between sociodemographic factors and knowledge about LF infection, prevention and control

Variables	Standard Error	Odds ratio	95% CI of Odds ratio		P-value
			Lower	Upper	
Age					
≤30 years		1			
31-40 years	0.267	1.304	0.772	2.202	0.321
41-50 years	0.285	1.766	1.011	3.085	0.046
51-60 years	0.412	1.737	0.774	3.896	0.180
Sex					
Male	0.249	1.608	0.988	2.618	0.056
Female		1			
Profession					
Doctor		1			
Nurse	0.271	2.392	1.405	4.072	0.001
Pharmacist	0.687	2.824	0.735	10.852	0.131
Laboratory Scientist	0.576	4.886	1.580	15.107	0.006
Others^*^	0.457	6.069	2.477	14.870	0.000

* Radiographer, radiologist, pharmacy technician, community health extension worker, physiotherapist, laboratory technician, dental technician, nutritionist

**Table 5:** multivariate analysis to determine the association between sociodemographic factors and attitude of respondents about LF infection, prevention and control

Variables	Standard Error	Odds ratio	95% CI of Odds ratio		P-value
			Lower	Upper	
**Age**					
≤30 years		1			
31-40 years	0.314	0.144	0.510	1.746	0.854
41-50 years	0.334	1.242	0.645	2.391	0.517
51-60 years	0.448	0.477	0.199	1.148	0.099
**Profession**					
Doctor	0.483	2.654	1.029	6.845	0.043
Nurse	0.480	1.945	0.760	4.979	0.105
Pharmacist	1.152	10.093	1.055	96.516	0.045
Laboratory Scientist	0.783	1.296	0.279	6.014	0.740
Others^#,/sup>^		1			
**Department**					
Adult accident and emergency		1			
Infectious ward and community health	0.665	5.504	1.494	20.273	0.010
Diagnostic^*^	0.725	4.574	1.105	18.939	0.036
Obstetrics and gynaecology	0.891	18.209	3.177	104.372	0.001
Paediatrics	0.624	3.083	0.908	10.465	0.071
Medicine^**^	0.572	2.925	0.953	8.976	0.061
Surgery^***^	0.571	3.071	1.003	9.402	0.049
Other Clinical^****^	0.539	1.884	0.655	5.419	0.240

# Radiographer, radiologist, pharmacy technician, community health extension worker, physiotherapist, laboratory technician, dental technician, nutritionist ^*^diagnostic department: laboratory and radiology ^**^Medicine: family medicine, staff clinic and medicine ^***^Surgery: orthopaedics, ENT, ophthalmology, surgery. ^****^Other clinicals: anaesthesia, dental, psychiatry, pharmacy, physiotherapy, house officers

**Table 6:** multivariate analysis to determine the association between sociodemographic factors and practice of health workers regarding LF infection, prevention and control

Variables	Standard Error	Odds ratio	95% CI of odds ratio		P-value
			Lower	Upper	
**Age**					
≤30 years		1			
31-40 years	0.319	0.636	0.340	1.189	0.156
41-50 years	0.379	0.262	0.125	0.550	0.000
51-60 years	0.538	0.508	0.177	1.459	0.208
**Sex**					
Male		1			
Female	0.309	0.715	0.391	1.311	0.278
Profession					
Doctor	0.817	8.282	1.670	41.065	0.010
Nurse	0.843	4.605	0.882	24.036	0.070
Pharmacist	1.093	8.755	1.028	74.531	0.047
Laboratory scientist	1.298	0.609	0.048	7.748	0.702
Others^#^		1			
**Department**					
Adult accident and emergency		1			
Infectious ward and community health	0.990	0.412	0.059	2.867	0.370
Diagnostic*	0.832	4.631	0.907	23.638	0.065
Obstetrics and gynaecology	0.860	0.970	0.180	5.230	0.971
Paediatrics	0.747	4.097	0.947	17.722	0.059
Medicine^**^	0.749	1.425	0.328	6.186	0.636
Surgery^***^	0.713	3.105	0.768	12.546	0.112
Other clinicals^****^	0.690	2.726	0.703	10.540	0.146

# Radiographer, radiologist, pharmacy technician, community health extension worker, physiotherapist, laboratory technician, dental technician, nutritionist. ^*^Diagnostic department: laboratory and radiology. ^**^Medicine: family medicine, staff clinic and medicine. ^***^Surgery: orthopaedics, ENT, ophthalmology, surgery ^****^Other clinicals: anaesthesia, dental, psychiatry, pharmacy, physiotherapy, house officers

## Discussion

In this study, more than half (52.3%) of the health workers had appropriate knowledge of IPC measures for LF. This finding is the same as the findings from studies carried out among health workers in tertiary institutions [8,17]. A similar knowledge level was found in a study conducted on the knowledge of Crimean-Congo fever among healthcare workers in Iran where only an average of persons was knowledgeable [18]. The high level of appropriate knowledge could be due to the use of tertiary institutions and health facilities with a specialty in LF management as study areas. The finding in the present study shows a difference from studies conducted among local government health workers in Ondo and Edo States [14,15,19]. These studies recorded a low knowledge level of the prevention and control of LF infection. The disparity of our findings with these studies could be due to the higher level of educational qualification of the doctors, nurses, pharmacists, and laboratory scientists, unlike primary care workers. In this study, 92.7% of our respondents had tertiary education. Due to the high knowledge of LF displayed, the present study therefore reveals the appropriateness of doctors, nurses, and other allied health professionals in communicating the prevention and control strategies of LF to individuals.

A significant knowledge level was displayed by doctors and other frontline health workers in this study and this has been similarly reported in literature [19]. The display of a high level of knowledge among these professionals could be due to their high level of training and exposure previously. This could also be due to the saddled responsibility for aptness in handling any health event especially among doctors and nurses who made up over half of the respondents in this study. Our findings in this regard are in contrast to other studies where the knowledge of LF infection had no relationship with medical professionalism [20]. Our findings, therefore, imply the need for improved efforts in communicating regular up-to-date LF prevention and control strategies to frontline health workers who are more likely to be involved in its dissemination. We recorded a higher risk perception for LF infection among doctors and nurses compared to other groups in the healthcare profession. This implies that doctors and nurses identified that they are at higher risks for LF infection because they are more likely to come in close contact with LF-patients than others. This finding is similar to the perception of healthcare workers in Edo State, Nigeria where a good risk perception for LF was observed [15]. The reference study also noted that community health workers (CHWs) had good risk perception for LF infection. The variance in these findings could be due to the unavailability of doctors or nurses in some health care settings, thereby making CHWs the only available source for information on the risk factors for LF infection.

The level of positive attitudes towards LF infection, prevention and control was low compared to the high level of positive attitudes found in studies carried among healthcare workers in some tertiary institutions in Nigeria [15,21]. Another study conducted in Ile-Ife similarly reported a low level of positive attitude towards LF IPC [16]. The findings from the present study are in tandem with findings from a study conducted among healthcare workers in both private and public health facilities in Edo State [15]. This finding implies that healthcare workers are expected to portray positive attitudes regarding LF IPC. There was a significant association between the department of the healthcare worker and the attitude towards LF IPC. A contrast was reported by a similar study where the designation of health workers was not associated with attitude regarding LF IPC [19]. The findings in the present study could have been informed by the training and retraining of healthcare workers in the department because the institution primarily cares for LF-positive patients. The positive attitude observed in this study is not therefore surprising among healthcare workers.

Regarding practices on LF IPC, we found that a higher proportion of HCWs had good preventive practices for LF infection. This finding was corroborated by a study conducted among adult residents of a rural community in Edo State, Nigeria where a low level of positive practice on the prevention and control of LF infection was observed [22]. A study conducted among residents of Abakaliki metropolis, Nigeria showed a higher proportion of respondents with good LF preventive practices [23]. This finding is however contrary to findings from a study conducted in a rural community in Nigeria where a low level of positive practices on the IPC measures for LF was recorded. The findings in the present study thus imply a higher practice of preventive measures for LF infection. We observed a significant relationship between the profession and department of the healthcare workers and their practice towards LF IPC. Previous studies have reported contrary findings that there is no relationship between the profession and department of HCWs and their practice of the prevention and control of LF infection [19]. This finding highlights the need for improved IPC training among healthcare workers, regardless of their expertise.

The availability of soap for hand washing, water, wash hand basin, and 0.05% chlorine solution, and gloves were the most frequently mentioned facilities and supplies by respondents. The mention of the use of gloves in personal protection was similarly cited among health workers in Edo State Nigeria, and nursing and midwifery students in Turkey [19,24]. Faceshield or goggles, 0.1% chlorine solution, recoded waste bins, apron, and full-body PPE were less frequently cited by respondents as available in the present study. The higher availability of soap, water, wash hand basin, and 0.05% chlorine solution could be due to the perceived essentiality of these PPE compared to others. Although according to standards, the World Health Organization has recommended the provision of standard personal protective gear including scrub suit, gown, aprons, rubber boots, face shield, face masks, and two pairs of gloves [19]. Unfortunately, the unavailability of some of these protective gears in health facilities due to limited finances makes the implementation of IPC measures difficult. This increasingly exposes health workers to the risk of LF infection. Emphasis should be laid on the increased availability of protective gear to health facilities.

## Conclusion

Healthcare workers are expected to display high levels of knowledge, and positive attitudes and practices regarding the prevention and control of LF in healthcare settings. To enhance the IPC measures among healthcare workers, we hereby recommend the prompt provision of IPC facilities and equipment in adequate amounts to health facilities. Training and retraining of HCWs should be intensified regularly to improve the adoption of a positive attitude regarding LF among them. Also, an assessment of the knowledge level of HCWs on their knowledge of LF risks, and its prevention and control measures should be done regularly. This would help to identify existing gaps in their knowledge base of LF and to be able to channel intervention strategies to address such gaps.

### What is known about this topic

- The control of LF infection is important and could be achieved through appropriate IPC knowledge, attitude and practice;
- Poor knowledge of IPC for LF among HCWs is unfortunate because HCWs are active agents of promoting compliance to disease preventive measures.

### What this study adds

- A knowledge-attitude-practice gap exists among health workers towards prevention and control of LF;
- An assessment of the knowledge, attitude, and practices of health workers is needed to identify existing gaps in their knowledge base of LF and to be able to channel intervention strategies to address such gaps;
- To enhance adherence to LF infection prevention, and control practices, training, and retraining of health workers should be intensified regularly to improve the adoption of a positive attitude regarding LF among them.
